# Supplementary material for: Y-linked editors for invasive rodent control
Source: Mol Biol Evol. 2026 Jul 23;43(7):msag165. doi: 10.1093/molbev/msag165 (PMC13392571; doi:10.1093/molbev/msag165)
Supplement: msag165_Supplementary_Data [file msag165_supplementary_data.pdf]

Y-linked editors for invasive rodent control

S1 Text: Supplemental analyses, figures and tables

Prateek Verma[1], Omar S. Akbari[†2], John M. Marshall[†1,3]

[1]*Divisions of Biostatistics & Epidemiology, School of Public Health, University of California, Berkeley, California, United States of America*

[2]*School of Biological Sciences, Department of Cell and Developmental Biology, University of California, San Diego, California, United States of America*

[3]*Innovative Genomics Institute, Berkeley, California, United States of America*

Supplementary figures

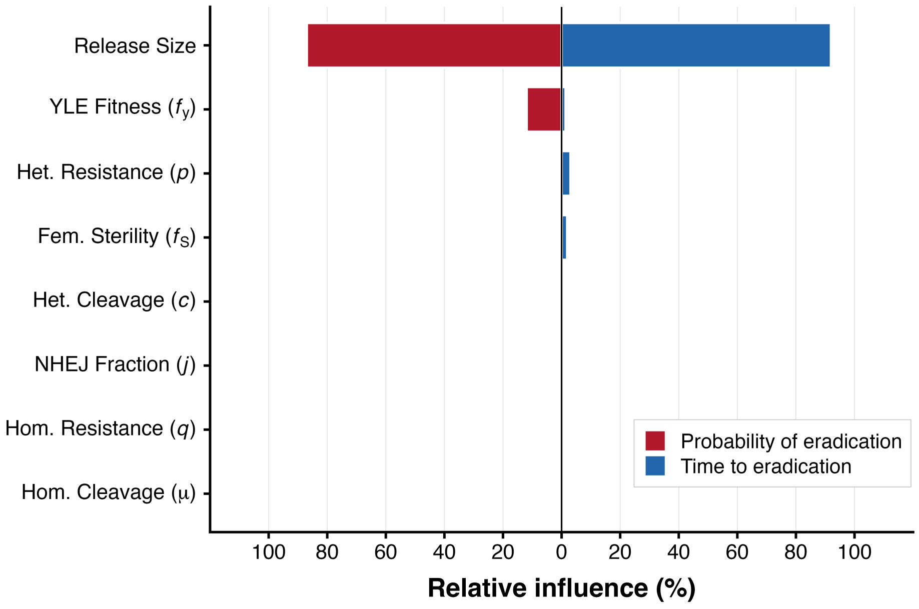

Fig. A: Relative influence of input parameters on eradication outcomes based on boosted regression tree (BRT) analysis. The influence of seven model parameters on two distinct outcomes was evaluated: the likelihood of achieving successful eradication (probability of eradication) and, among successful simulations, the time required to reach eradication (time to eradication). Bar values represent the relative contribution (percentage) of each parameter to the predictive performance of the respective BRT models. The influence scores sum to 100% for each outcome separately. Parameters with larger values have a greater impact on the model's predictions for that specific outcome.

*Correspondence: oakbari@ucsd.edu, john.marshall@berkeley.edu

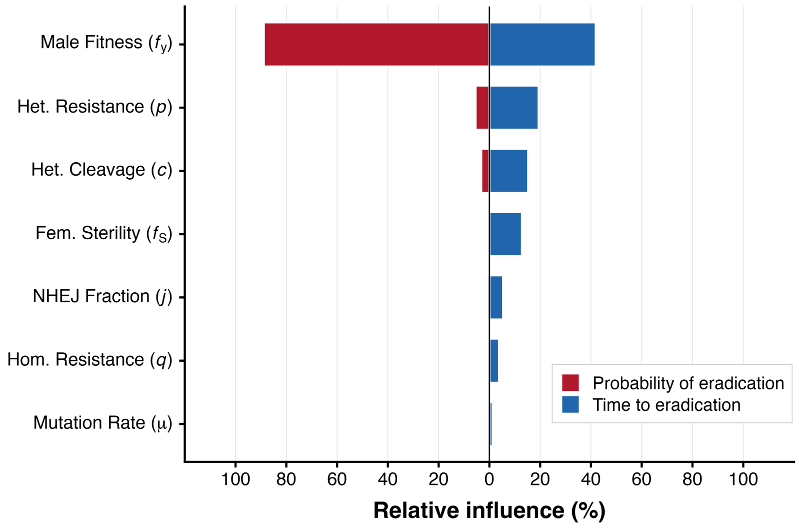

Fig. B: Relative influence of input parameters with fixed release size. BRT analysis of parameter contributions to eradication probability and time to eradication. Here, the relative release size was held constant at 3% of adult male carrying capacity. All other parameter ranges and modeling definitions are identical to Fig. A.

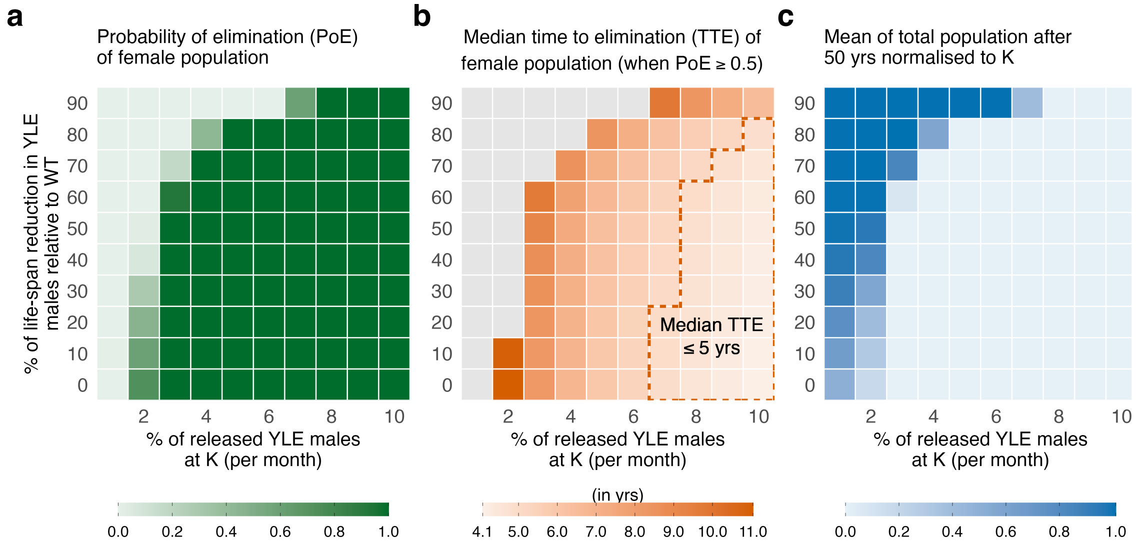

Fig. C: Performance of a Y-linked editor (YLE) targeting a female-specific dominant lethality gene over a 10-year release period. Each panel maps outcomes over a common parameter grid where the x-axis is the release proportion of YLE males (percent of adult males at carrying capacity, $K = 10{,}000$), released monthly for 10 years, and the y-axis is the fitness cost to YLE carriers measured as percent reduction in lifespan. For each parameter combination, 100 stochastic simulations were performed. Panel (a) shows the probability of female elimination (0–1). Panel (b) shows the median time to female elimination (in years), computed only when the elimination probability is $\geq 50\%$. Darker orange indicates higher time to elimination, and grey indicates elimination probability $< 50\%$. The dashed orange perimeter marks parameter combinations where the median time to elimination is ≤ 5 years. Panel (c) shows the final total population after 50 years, normalized with respect to K and averaged across runs (0–1 scale).

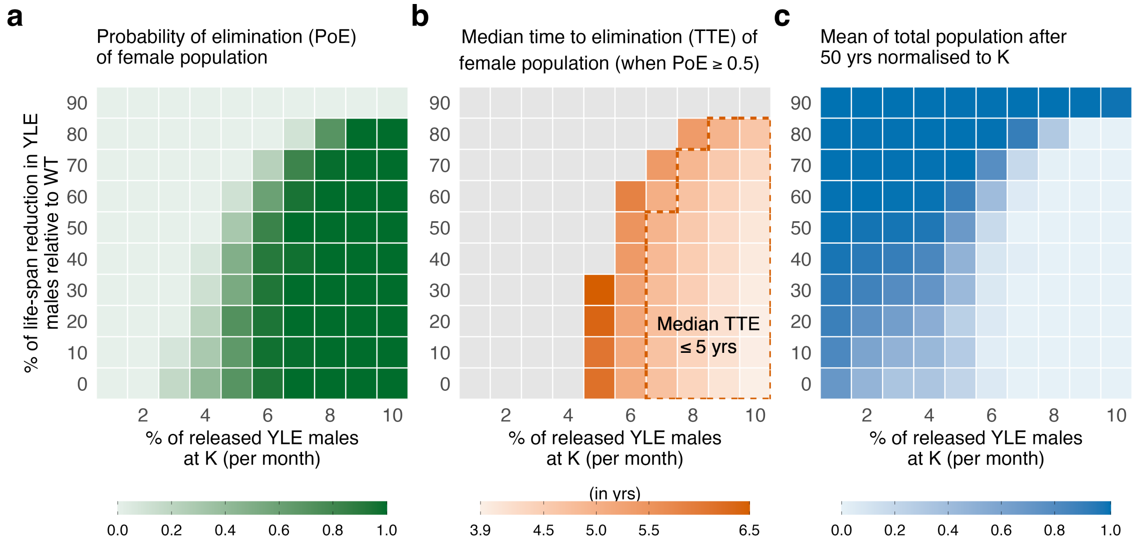

Fig. D: Performance of a Y-linked editor (YLE) targeting a female-specific dominant lethality gene over a 5-year release period. Each panel maps outcomes over a common parameter grid where the x-axis is the release proportion of YLE males (percent of adult males at carrying capacity, K = 10,000), released monthly for five years, and the y-axis is the fitness cost to YLE carriers measured as percent reduction in lifespan. For each parameter combination, 100 stochastic runs were performed. Panel (a) shows the probability of female elimination (0–1). Panel (b) shows the median time to female elimination (in years), computed only when the elimination probability is $\geq$ 50%. Darker orange indicates higher time to elimination, and grey indicates elimination probability $<$ 50%. The dashed orange perimeter marks parameter combinations where the median time to elimination is $\leq$ 5 years. Panel (c) shows the final total population after 50 years, normalized with respect to K and averaged across runs (0–1 scale).

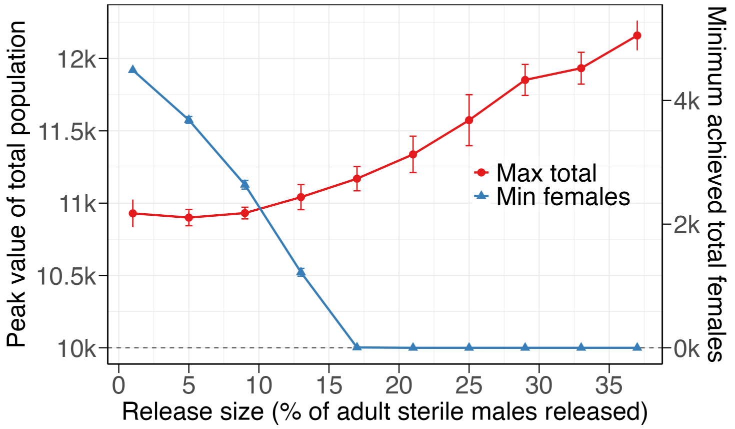

Fig. E: Suppression efficacy and population peaks as a function of release size for release of rodents carrying a female-specific dominant lethal gene (fsRRDL). Mean peak total population (red) and minimum wild-type female population (blue) achieved during a ten-year fsRRDL campaign are shown as a function of monthly release size. The dashed dark grey line represents the equilibrium population size in the absence of interventions (K = 10,000). Data represent means from 100 stochastic runs.

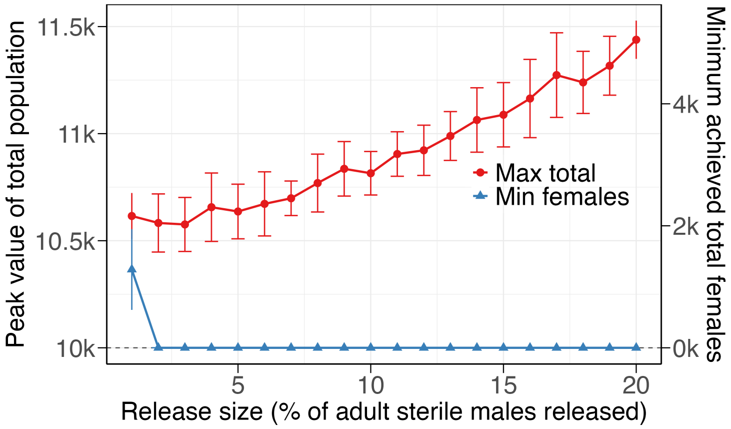

Fig. F: Suppression efficacy and population peaks as a function of release size for a Y-linked editor (YLE). Mean peak total population (red) and minimum wild-type female population (blue) achieved during a ten-year YLE campaign are shown as a function of monthly release size. The dashed dark grey line represents the equilibrium population size in the absence of interventions ($K = 10{,}000$). Data represent means from 100 stochastic runs (baseline parameters: Table B).

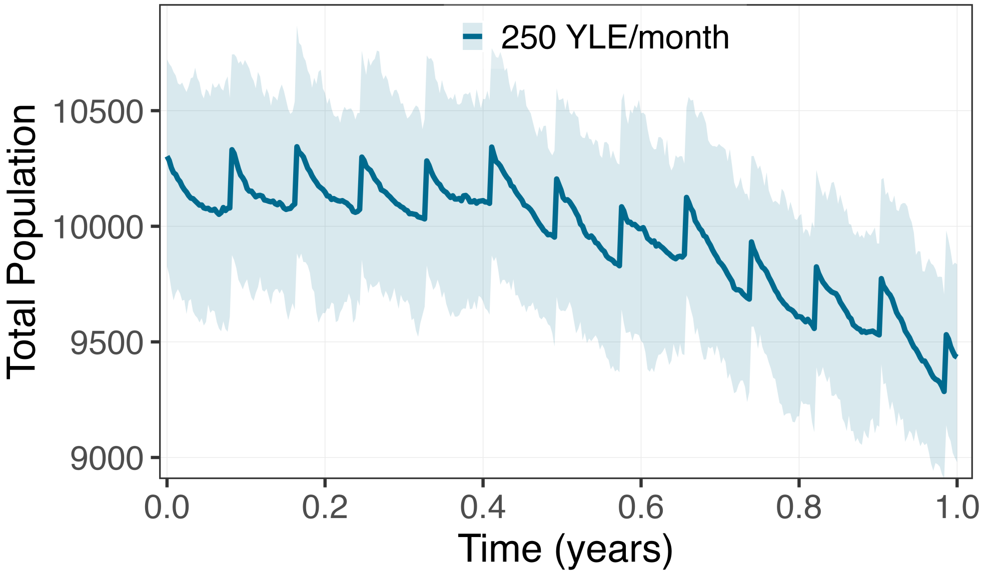

Fig. G: Transient population overshoot during the initial phase of the Y-linked editor (YLE) suppression campaign. The total mouse population trajectory is shown for the first year of intervention involving continuous monthly releases. A minor transient increase of approximately 3% above the equilibrium capacity ($K = 10{,}000$) is observed during the first nine releases. This temporary rise persists for approximately 35 weeks before the population drops below baseline levels and suppression takes effect.

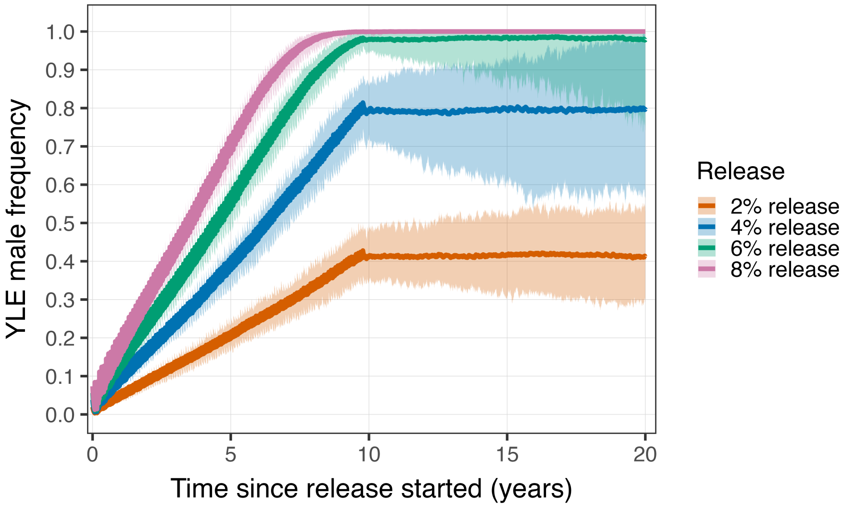

Fig. H: Temporal dynamics of Y-linked editor (YLE) frequency in males for a haplosufficient (recessive) female-sterile target under varying release sizes. Bold colored lines show the mean YLE male frequency across stochastic simulations, with shaded ribbons of 95% confidence interval across 100 simulation replicates. Releases were conducted monthly for ten years, with release sizes ranging from 2% to 8% of the baseline adult male population at carrying capacity ($K = 10000$). All simulations assume no fitness cost associated with the YLE construct and use baseline life-history parameters (Table A, S1 Text). At higher release sizes (6–8%), the YLE increases rapidly and approaches fixation within the release period.

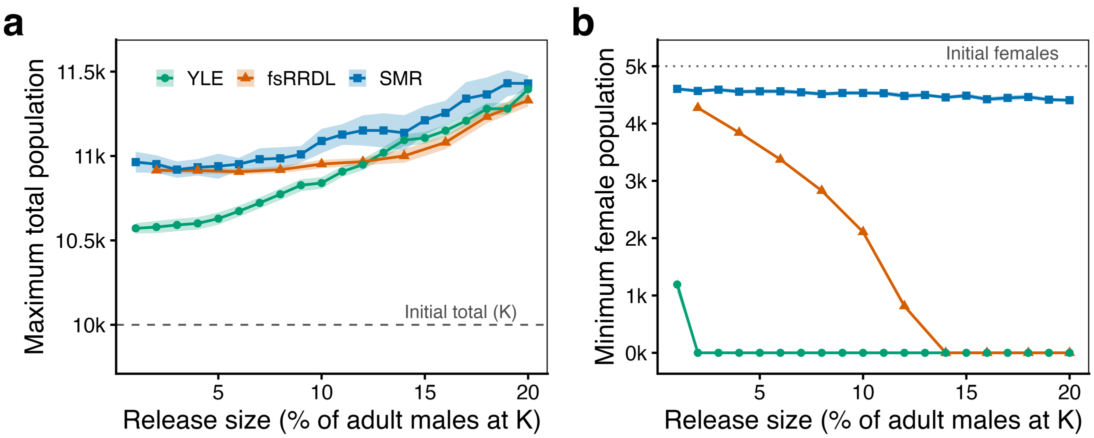

Fig. I: Comparison of suppression outcomes across release sizes for Y-linked editor (YLE), fsRRDL, and sterile male release (SMR) strategies. (a) Maximum total population size observed during the simulation period as a function of release size, expressed as a percentage of the adult male population at carrying capacity. The dashed line indicates the initial total population size ($K = 10{,}000$). (b) Minimum female population size achieved during the simulation period across the same range of release sizes. The dotted line indicates the theoretical initial female population size. Data represent means from 100 stochastic runs, with shaded ribbons indicate 95% confidence interval range around mean values.

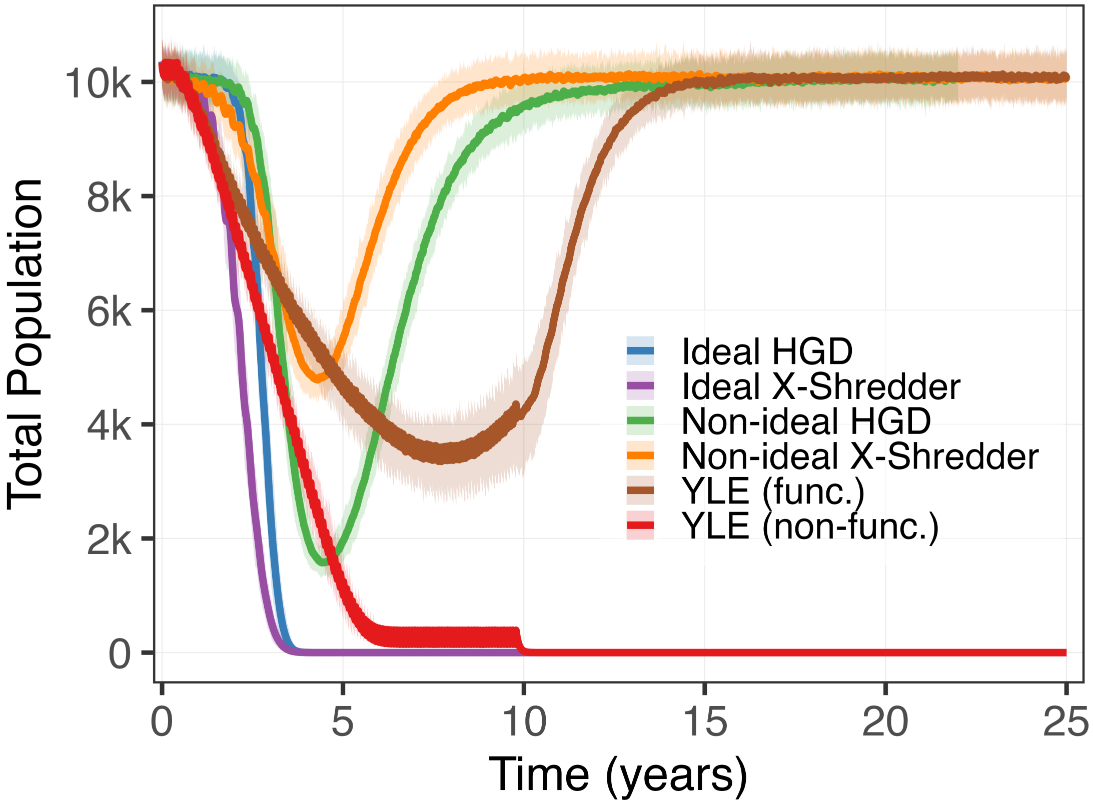

Fig. J: Impact of functional resistance on Y-linked editor (YLE) dynamics under repeated releases. Population trajectories for YLE strategies assuming non-functional resistance (recessive sterile; baseline) and functional resistance, compared alongside ideal and non-ideal homing gene drives (HGD) and X-shredder systems. The YLE strategy involves monthly releases of 250 adult males for ten years, while HGD and X-shredder strategies involve a single release of 250 males. Functional resistance assumes that resistant alleles preserve target gene function, whereas non-functional resistance assumes resistant alleles are recessively sterile. Under functional resistance, the YLE fails to achieve population elimination at this release size and the population rebounds following initial suppression, similar to other non-ideal drive scenarios. Lines show mean total population size and shaded regions represent 95% confidence intervals across 100 stochastic simulations. All simulations are conducted in a single isolated population at carrying capacity (K = 10,000), with parameter values as specified in Tables B, D, and E.

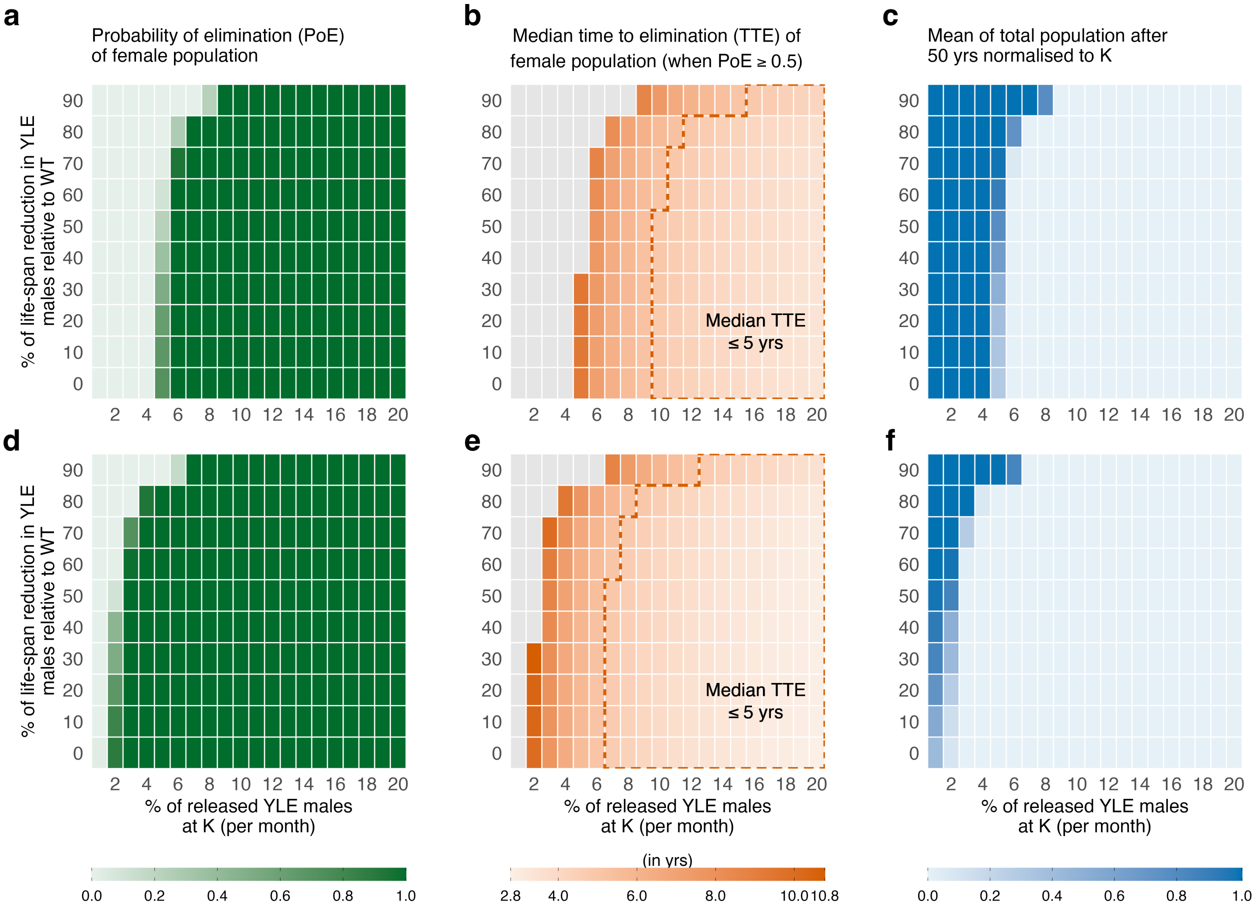

Fig. K: Impact of functional versus non-functional resistance on Y-linked editor (YLE) performance. (a–c) Probability of elimination (PoE), median time to elimination (TTE), and mean population size after 50 years (normalised to $K = 10{,}000$) as functions of monthly release size and reduction in male lifespan, assuming functional resistance. In this case, resistance alleles preserve target gene function (i.e., VV genotypes are viable and fertile), while edited alleles remain haploinsufficient. (d–f) Comparison of YLE performance under functional resistance and the baseline case with non-functional (recessively sterile) resistance, where $fXVV$ females are sterile. Functional resistance reduces suppression efficacy and increases the threshold release size required for elimination relative to the baseline case. Results are based on 100 stochastic simulations per parameter combination.

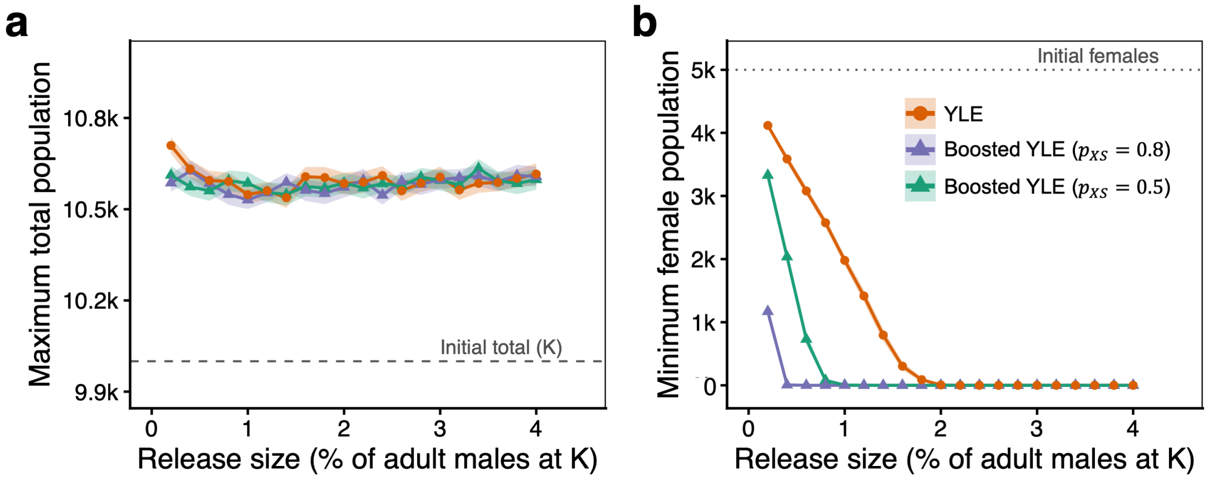

Fig. L: Transient dynamics of boosted Y-linked editor (YLE) strategies compared to the standard YLE. (a) Mean maximum total population size achieved during the release phase as a function of monthly release size for the standard YLE and boosted YLE configurations with X-shredder efficiencies $p_{XS} = 0.8$ and $p_{XS} = 0.5$. The dashed grey line indicates the initial population size at carrying capacity (K = 10,000). Across the range of release sizes considered, boosted YLE strategies do not produce a substantial increase in peak population size relative to the standard YLE. (b) Mean minimum wild-type female population size achieved during the release phase for the same strategies. Boosted YLE configurations achieve substantially deeper female suppression at lower release sizes compared to the standard YLE. The dotted grey line indicates the initial female population. Results are means across 100 stochastic simulations, with shaded regions 95% confidence inverval range around the mean line across runs.

Supplementary tables

Table A: Description and baseline values of mouse life history parameters used in the MouseGD framework. All parameter values are adopted from Brown et al. [1]. Derived demographic quantities based on these parameters include the basic reproductive number at low density ($R_m \approx 1.05$), the generation time at low density ($T_{\text{low}} \approx 109$ days), and the generation time at carrying capacity ($T_K \approx 98$ days)*.

**Parameter**	**Description**	**Baseline value**	**Units**
t_{Gest}	Gestation duration: average time females remain pregnant before parturition.	19	days
t_{Nurs}	Nursing duration: time offspring remain dependent on the dam post-birth.	23	days
t_{Ado}	Adolescence duration: time juveniles remain pre-reproductive before entering the adult stage.	37	days
t_{Ad}	Mean residence time in the adult stage; determines baseline daily adult mortality.	690	days
μ_{Ad}	Density-independent adult mortality applied each day to adults.	1/690	per day
L	Effective average number of litters produced per adult female per year; maps to a daily conception probability.	7.5	litters/female/yr
Q	Mean effective offspring per litter (surviving to adulthood).	6	pups per litter
$K^{\dagger}$	Adult carrying capacity; regulates the intensity of density-dependent adult mortality.	10,000	Adults #
$\Theta^{\ddagger}$	Shape parameter of density dependence; steeper values increase mortality as adults approach or exceed K.	22.4	Unitless
M	Mating system: females mate once per reproductive cycle with a randomly selected male; no multiple mating or assortative mating is assumed.	Single mating	NA

† K represents the adult carrying capacity.
‡ Θ is the shape parameter for density-dependent adult mortality, defined by daily probability of adult survival, $s(A_d)$:

$$s(A_d) = (1 - \mu_{Ad}) \left(\frac{0.5\,K}{K + A_d} \right)^{\Theta},$$

see Eq. 1, Methods Section 4.2 of the main text.
* The derived quantities R_m, T_{low}, and T_K are obtained using an Euler–Lotka formulation based on the life-history parameters listed here. The full derivation is provided in the subsequent section of this Supplementary Text (S1 Text).

Table B: Model parameters for Y-linked editor (YLE) inheritance, fitness, and release strategies. The table lists baseline values used for primary simulations alongside the parameter ranges used for global sensitivity analysis (SA). $U(a, b)$ denotes a continuous uniform distribution on the interval $[a, b]$. Literature sources for specific parameter estimates are provided in the reference column.

Parameter	Description	Baseline value	SA distribution	Reference
μ	Per-allele cleavage rate in yWW males during gametogenesis, representing edit initiation in homozygotes.	0.97	$U(0.80, 1.00)$	[2]
q	In yWW males after cleavage, the fraction of repairs that yield a resistant or recessive variant allele, V; the remaining fraction, $1 - q$, yields the desired edited allele, E.	0.00	$U(0.00, 0.20)$	[3]
c	Cleavage rate of a wild-type allele, W, in heterozygous males (yWE or yWV), representing edit initiation in heterozygotes.	0.93	$U(0.80, 1.00)$	[2]
j	Given cleavage in heterozygotes, the rate of non-homologous end joining (NHEJ), relative to homology-directed repair (HDR). With HDR, $1 - j$, the W allele copies the homologous allele, either E or V.	0.25	$U(0.00, 1.00)$	[4, 5]
p	In heterozygotes after NHEJ, the rate at which the NHEJ outcome is a resistant or recessive lethal variant allele, V, rather than the desired edited allele, E, which occurs with probability $1 - p$.	0.00	$U(0.00, 0.20)$	[3]
f_y	Relative fitness of YLE males with respect to wild-type males, implemented as a reduction in expected lifespan.	0.8	$U(0.20, 1.00)$	[3]
f_S	Expressivity of female sterility with one or more E alleles, where 0 denotes no effect and 1 denotes complete sterility.	1 (0 if viability-targeted)	$U(0.80, 1.00)$	[2]
f_L	Expressivity of female lethality with one or more E alleles, where 0 denotes no effect and 1 denotes complete lethality.	0 (1 if viability-targeted)	$U(0.80, 1.00)$	[2]
rel	The ratio of released yEE males to the baseline adult male population at K, per monthly release event	Scenario-specific	$U(0.01, 0.20)$	–

Table C: Relative influence of model parameters on eradication outcomes. Sensitivity analysis was performed using boosted regression trees (BRTs) to evaluate the impact of input parameters on two metrics: the probability of successful population eradication and the time to eradication (conditioned on success). Values represent the relative contribution (%) of each parameter to the predictive power of the BRT models. Parameters are sorted in descending order of their influence on the probability of eradication.

Parameter	Description	Rel. Influence (Probability of Elimination)	Rel. Influence (Time to Elimination)
rel	Release size	86.99%	91.98%
f_y	YLE fitness	11.95%	1.38%
p	Heterozygous resistance	0.56%	3.06%
c	Heterozygous cleavage	0.18%	0.57%
q	Homozygous resistance	0.11%	0.40%
μ	Homozygous cleavage	0.09%	0.09%
f_S	Female sterility	0.08%	1.92%
j	NHEJ fraction	0.04%	0.60%

Table D: Drive mechanics and release parameters for homing gene drives (HGDs). Parameter sets are distinguished for ideal scenarios (assuming perfect homing and no resistance) and non-ideal scenarios (incorporating observed rates of non-homologous end-joining (NHEJ) repair and formation of functional resistance alleles). Sources for parameter values are listed in the reference column.

**Parameter**	**Description**	**Ideal condition**	**Non-ideal condition**	**Reference**
p_C	Rate that the target allele is cut in a germline cell (cutting efficiency).	1.00	0.97	[2]
p_N	Rate of NHEJ repair given that a cut occurs.	0.00	0.25	[4, 5]
p_L	Fraction of NHEJ events that generate a loss-of-function (disruptive) allele.	1.00	5/6	[6, 7]
f_W	Relative fitness of wild-type allele (W).	1.00	1.00	[3]
f_H	Relative fitness of homing drive allele (H).	1.00	0.80	[3]
f_R	Relative fitness of functional resistant allele (R).	1.00	1.00	[3]
f_N	Relative fitness of non-functional allele (N) carriers with respect to wild-type as reduction in expected lifespan.	0.00	0.50	–
rel	Released adult males (yHH) introduced (single release).	250	250	–

Table E: Drive mechanics and release parameters for Y-linked X-shredder systems. Values define ideal performance (perfect sex-ratio distortion) versus non-ideal performance (accounting for incomplete shredding efficiency and associated fitness costs). Relevant literature sources for these values are cited in the reference column.

Parameter	Description	Ideal condition	Non-ideal condition	Reference
c_X	Probability that an X chromosome is successfully shredded in X-shredder males.	1.00	0.97	[2]
c_{rX}	Rate of resistance chromosome generation.	0.00	0.25	[4, 5]
f_{XA}	Relative fertility fitness of males carrying the attacking Y chromosome or X-shredder construct (mXA, mRA).	1.00	0.80	[3]
rel	Released adult males (mXA) introduced (single release).	250	250	–

Basic reproductive number and generation time

We derive the basic reproductive number, R_m, and generation time using the Euler–Lotka framework based on the life-history parameters in Table A.

Life-cycle timing and fecundity

Age at maturity and first reproduction are

$$A_{\text{mat}} = t_{\text{Nurs}} + t_{\text{Ado}} = 60 \text{ days}, \qquad A_0 = A_{\text{mat}} + t_{\text{Gest}} = 79 \text{ days}.$$

Daily female offspring production is

$$f = \frac{L}{365} \cdot \frac{Q}{2} = 0.06164 \text{ female offspring per female per day}.$$

Adult survival

Daily adult survival is

$$s(A_d) = (1 - \mu_{Ad}) \left(\frac{K/2}{K + A_d} \right)^{1/\Theta}.$$

At low density ($A_d \to 0$),

$$s_{\text{low}} = \left(1 - \frac{1}{690}\right) \left(\frac{1}{2}\right)^{1/\Theta} = 0.96812. \tag{1}$$

At carrying capacity ($A_d = K$),

$$s_K = \left(1 - \frac{1}{690}\right) \left(\frac{1}{4}\right)^{1/\Theta} = 0.93863. \tag{2}$$

Basic reproductive number

The basic reproductive number is

$$R_m = \sum_{a=A_0}^{\infty} \ell(a) m(a),$$

with

$$\ell(a) = s_{\text{low}}^{a - A_{\text{mat}}}, \qquad m(a) = f.$$

Thus

$$R_m = f s_{\text{low}}^{A_0 - A_{\text{mat}}} \sum_{k=0}^{\infty} s_{\text{low}}^k = \frac{f s_{\text{low}}^{A_0 - A_{\text{mat}}}}{1 - s_{\text{low}}}.$$

$$\boxed{R_m = \frac{f s_{\text{low}}^{19}}{1 - s_{\text{low}}} = 1.045} \tag{3}$$

Euler–Lotka equation

The intrinsic growth rate r satisfies

$$f s^{A_0 - A_{\text{mat}}} \frac{e^{-rA_0}}{1 - s e^{-r}} = 1. \tag{4}$$

Generation time

Generation time is

$$T = \sum_{a=0}^{\infty} a\, e^{-ra} \ell(a) m(a). \tag{5}$$

This reduces to

$$T = f s^{A_0 - A_{\mathrm{mat}}} e^{-rA_0} \left[\frac{A_0}{1 - se^{-r}} + \frac{se^{-r}}{(1 - se^{-r})^2} \right]. \tag{6}$$

Low-density conditions

Using $s_{\mathrm{low}} = 0.96812$ from Eq. (1) and solving Eq. (4),

$$r_{\mathrm{low}} = 0.000403\ \mathrm{day}^{-1}.$$

$$\boxed{T_{\mathrm{low}} = 108.99\ \mathrm{days} = 0.299\ \mathrm{years}} \tag{7}$$

Carrying capacity

Using $s_K = 0.93863$ from Eq. (2) and solving Eq. (4),

$$r_K = -0.01248\ \mathrm{day}^{-1}.$$

$$\boxed{T_K = 98.17\ \mathrm{days} = 0.269\ \mathrm{years}} \tag{8}$$

Comparing T_{low} and T_K, the generation time decreases with increasing density because density-dependent mortality reduces adult survival. This effectively reduces the mean age of the parents of the newborn at carrying capacity compared to the low density limit.

References

[1] Brown EA, Eikenbary SR, Landis WG. Bayesian network-based risk assessment of synthetic biology: Simulating CRISPR-Cas9 gene drive dynamics in invasive rodent management. *Risk Anal.* 2022;42:2835–2846.

[2] Douglas C, Maciulyte V, Zohren J, Snell DM, Mahadevaiah SK, Ojarikre OA, et al. CRISPR-Cas9 effectors facilitate generation of single-sex litters and sex-specific phenotypes. *Nat Commun.* 2021;12:6926.

[3] Tolosana I, Willis K, Gribble M, Phillimore L, Burt A, Nolan T, et al. A Y chromosome-linked genome editor for efficient population suppression in the malaria vector *Anopheles gambiae*. *Nat Commun.* 2025;16:206.

[4] Grunwald HA, Gantz VM, Poplawski G, Xu X-RS, Bier E, Cooper KL. Super-Mendelian inheritance mediated by CRISPR-Cas9 in the female mouse germline. *Nature.* 2019;566:105–109.

[5] Birand A, Gierus L, Prowse TAA, Cassey P, Thomas PQ. Maximising eradication potential of rat gene drives using a two-target homing rescue strategy: Spatial modelling of empirical data. *Mol Ecol.* 2025;34:e17777.

[6] Adolfi A, Gantz VM, Jasinskiene N, Lee H-F, Hwang K, Terradas G, et al. Efficient population modification gene-drive rescue system in the malaria mosquito *Anopheles stephensi*. *Nat Commun*. 2020;11:5553.

[7] Carballar-Lejarazú R, Pham TB, Tushar T, James AA. Mutant allele formation and inheritance during Cas9/guide RNA-mediated gene drive in a population modification mosquito strain for human malaria control. *Genetics*. 2025;231:iyaf176.
